# Development of a Weight Loss Mobile App Linked With an Accelerometer for Use in the Clinic: Usability, Acceptability, and Early Testing of its Impact on the Patient-Doctor Relationship

**DOI:** 10.2196/mhealth.4546

**Published:** 2016-03-31

**Authors:** Seryung Choo, Ju Young Kim, Se Young Jung, Sarah Kim, Jeong Eun Kim, Jong Soo Han, Sohye Kim, Jeong Hyun Kim, Jeehye Kim, Yongseok Kim, Dongouk Kim, Steve Steinhubl

**Affiliations:** ^1^ Department of Family Medicine Seoul National University Bundang Hospital Seongnam Republic Of Korea; ^2^ Department of Digital Medicine Scripps Translational Science Institute La Jolla, CA United States; ^3^ Department of Health Promotion Center Seoul National University Bundang Hospital Seongnam Republic Of Korea; ^4^ Department of Medical Nutrition Graduate School of East West Medical Science Kyung Hee University Yongin Republic Of Korea; ^5^ Department of Mental Health and Behavioral Medicine Services for Clinical Departments Seoul National University Bundang Hospital Seongnam Republic Of Korea; ^6^ INFINITT Healthcare Co., Ltd. Seoul Republic Of Korea

**Keywords:** mobile apps, electronic health record, weight reduction programs, physician-patient relations

## Abstract

**Background:**

Although complications of obesity are well acknowledged and managed by clinicians, management of obesity itself is often difficult, which leads to its underdiagnosis and undertreatment in hospital settings. However, tools that could improve the management of obesity, including self-monitoring, engagement with a social network, and open channels of communication between the patient and doctor, are limited in a clinic-based setting.

**Objective:**

The objective of our study was to evaluate the usability and acceptability of a newly developed mobile app linked with an accelerometer and its early effects on patient-doctor relationships.

**Methods:**

From September 2013 to February 2014, we developed a mobile app linked with an accelerometer as a supportive tool for a clinic-based weight loss program. The app used information from electronic health records and delivered tailored educational material. Personal goal setting, as well as monitoring of weight changes and physical activity combined with feedback, are key features of the app. We also incorporated an interactive message board for patients and doctors. During the period of March 2014 to May 2014, we tested our mobile app for 1 month in participants in a hospital clinic setting. We assessed the app’s usability and acceptability, as well as the patient-doctor relationship, via questionnaires and analysis of app usage data.

**Results:**

We recruited 30 individuals (18 male and 12 female) for the study. The median number of log-ins per day was 1.21, with the most frequently requested item being setting goals, followed by track physical activities and view personal health status. Scales of the depth of the patient-doctor relationship decreased from 27.6 (SD 4.8) to 25.1 (SD 4.5) by a Wilcoxon signed rank test (*P*=.02).

**Conclusions:**

A mobile phone app linked with an accelerometer for a clinic-based weight loss program is useful and acceptable for weight management but exhibited less favorable early effects on patient-doctor relationships.

## Introduction

In South Korea, management of obesity remains challenging within the current health care system [1,2] despite the existence of numerous guidelines [3-5] and effective treatments [6,7]. Although the consequences of obesity, such as hypertension, type 2 diabetes, or knee osteoarthritis, are promptly evaluated and managed by clinicians in the hospital setting, the core disease itself is frequently underdiagnosed and undertreated. A retrospective analysis [8] found significant disparities between the reported and true prevalence of obesity in outpatient departments, such as orthopedics (3% vs 25.1%), cardiology (4% vs 30%), and rheumatology (5% vs 20.2%).

Given obesity’s negative impact on premature death [9], socioeconomic costs [10-12], and quality of life [13], it is important to manage obesity in hospital settings via a coordinated and multidisciplinary approach with a comprehensive lifestyle program [3] that includes implementing a low-calorie diet, increasing physical activities through the use of behavioral strategies, and providing additional support from pharmacotherapy or surgical treatment in certain patients [14]. However, there are several limitations to managing obesity in a clinic-based setting. From a patient’s perspective, clinic visits are time consuming and costly, and these problems are exacerbated by inadequate patient-doctor interaction time. Behavioral intervention strategies [15], including self-monitoring of weight, diet, and physical activities, are key components of successful weight management. However, the tools required for self-monitoring and interactive feedback with clinicians are often limited in a clinic-based setting. Educating patients [16] concerning the risk of obesity and their active participation remains the cornerstone of weight loss programs, which require a repetitive and considerable effort by the clinicians. Moreover, a patient’s social network, social influence, and social support [17,18] are important aspects to consider, and these factors are typically lacking in the clinical management of obesity.

Recently, given the exponential spread of mobile phones, the Internet and mobile apps have become widely accessible, anytime and anywhere. Mobile apps in health care [19] have been regarded as a potential tool for altering patients’ behavior and improving pretreatment regimens.

Particularly in a clinic-based weight loss program, a mobile phone app can be a great resource for both patients and clinicians. To a patient, self-monitoring and self-regulation via a mobile app may be a more effective method than a paper-based monitoring system [20,21]. A mobile app can also serve as a tailored and customized educational tool for individualized services [22] and as a social support and social engagement tool [23] for weight loss. Considering the positive association [24] between the patient-doctor relationship and treatment adherence, with better patient satisfaction and outcomes [25-27], a mobile app has the potential to strengthen the patient-doctor relationship by making clinicians more focused on the patient’s concerns and consequently spending less time obtaining or providing basic information. In general, patients are more likely to be actively involved in the management of their disease if they perceive that they are engaged in a good-quality interpersonal exchange and have increased out-of-office contact with their physician [24]. Moreover, a mobile app may allow patients to play a more active role in the medical decision-making process, thus minimizing the knowledge gap between physicians and patients [27].

Despite an enormous number of available health care apps, most apps are underused [19], with limited approaches for user engagement, limited adoption of evidence-based behavioral-change strategies or frameworks [28], and limited evidence of clinically significant benefits derived from using the app [29]. Results of previous studies of a mobile app for weight loss were controversial regarding weight changes. One study of a mobile phone app for intervention [30] compared weight changes over 6 months between mobile app users and a control group. This study did not find significant weight loss in the intervention group, and a 42% dropout rate was noted within the first month. Another pilot study assessed a mobile phone app with a wearable monitoring device and compared the use of the app with a health education control group [31]. A significant meaningful weight reduction was noted in the monitoring device group.

Only a few studies have examined the use of mobile apps in hospital- or clinic-based weight loss programs and assessed how these apps may have affected the patient-doctor relationship. In our study, given that the app was developed for supportive use in a clinic-based weight loss program, we decided to link an accelerometer to the app. Therefore, our study aims were to develop a mobile app linked with an accelerometer for a clinic-based weight loss program and to test its usability, acceptability, and early effects on the patient-doctor relationship.

## Methods

### Development Process of the Mobile App Linked With an Accelerometer

One aim of this study was to describe the development process of a mobile app linked with an accelerometer for a clinic-based weight loss program. This study was part of a project to develop a personalized media service using a device-cloud interconnection for improving patient-doctor communication, which was funded by a grant from the Korean Evaluation Institute of Industrial Technology Research Fund. The development of the mobile app was planned and organized by a multidisciplinary team, including a team from Xeron Healthcare Corporation (Seoul, Republic of Korea), app designers, and teams from the hospital staff (family physicians and dietitians with a research coordinator; Seoul National University Bundang Hospital, Seongnam, Republic of Korea) between September 2013 and February 2014.

#### Theoretical Basis

The theoretical concept for this mobile app was mainly derived from social cognitive theory [32], which has played an important role in many weight loss clinical trials [33]. Weight management involves numerous lifestyle changes, including diet and exercise, which must be sustained for a long time. For such changes to be achieved and maintained over the course of an individual’s life, participants should believe in the value of weight loss and that their desired outcome is achievable through implementation of the required behavioral changes. Furthermore, those changes can be made through a key mediator [34]; self-regulation skills, such as goal setting, self-monitoring of behaviors (such as dietary intake or physical activities), or target outcomes (such as weight); and social support from important others [35].

Other important behavioral strategies have been noted to aid weight loss [36], such as time management, stimulus control, self-reward, relapse prevention, and emotion-focused strategies or cognitive strategies, which are all important components of successful weight management. However, the aim of our mobile app was to support weight management in clinic-based settings. Therefore, we adopted four main features in our app: (1) increased knowledge by providing personalized educational materials, (2) personal goal setting for weight loss, (3) self-monitoring of weight and physical activity linked with an accelerometer, and (4) online social support from a peer group as well as Web-based communication channels with their clinicians.

#### Definition of Obesity-Related Problems and Development of a Web-Based Interface Using Information From Electronic Health Records in a Clinic-Based Weight Reduction Program

**Figure 1:**
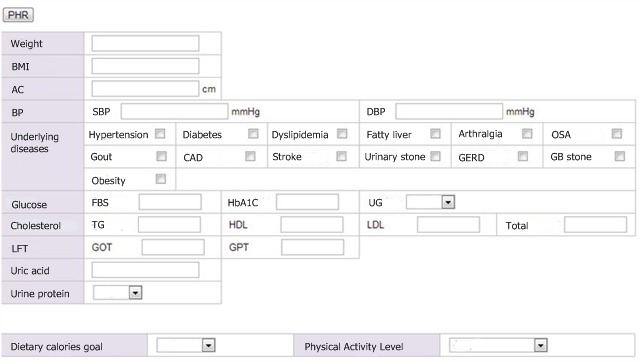
Screenshot of the Web-based interface through which the physician can input obesity-related information from the patient's electronic health record. AC: abdominal circumference; BMI: body mass index; BP: blood pressure; CAD: coronary artery disease; DBP: diastolic blood pressure; FBS: fasting blood sugar; GB: gallbladder; GERD: gastroesophageal reflux disease; GOT: glutamate oxaloacetate transaminase; GPT: glutamic pyruvate transaminase; HDL: high-density lipoprotein; LDL: low-density lipoprotein; LFT: liver function test; OSA: obstructive sleep apnea; PHR: patient health record; SBP: systolic blood pressure; TG: triglyceride; UG: urine glucose.

For the clinic-based weight loss program, we assessed patients and categorized their obesity-related problems [37,38] into subclinical states, symptomatic physical dysfunction, and established obesity-related diseases. We developed a Web-based interface for the mobile app to provide obesity-related information based on electronic health records (EHRs) to provide patients with tailored content and assessment tools, as Figure 1 shows. From EHRs we obtained anthropometric measurements, such as height, weight, age, abdominal circumference, and blood pressure, as well as laboratory measurements, including blood glucose, uric acid, triglyceride levels, high-density lipoprotein cholesterol, and low-density lipoprotein cholesterol. A family physician input information regarding obesity-related comorbidities, such as coronary artery disease, stroke, hypertension, diabetes, dyslipidemia, nonalcoholic steatohepatitis, arthritis, gout, obstructive sleep apnea, and reflux esophagitis, from the initial assessment. Participants consulted with dietitians and were prescribed a weekly sample menu of a low-calorie diet consisting of 1200 to 1800 kcal/day that was based on their age, sex, activity level, and target weight. Moreover, in accordance with the target weight and prescribed daily calories, participants were advised to achieve a target level of physical activity each day.

#### Delivery of Tailored Educational Materials Via a Web-Based Interface Using Information From EHRs

We considered the delivery of tailored educational materials about diseases and the specific weight loss target for each person as an important function of the app. We developed educational videos (see Multimedia Appendix 1 for the educational video for angina pectoris) for each obesity-related disease as part of the tailored educational materials. The educational videos featured a fictional scenario pertaining to a patient and an explanation of the mechanisms of the disease, as well as possible suggestions for weight loss. We set a target for the total running time for each video to be no greater than 1 minute and 30 seconds.

**Figure 2:**
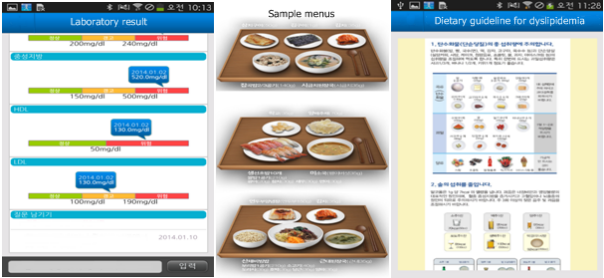
My Health Diary screenshot: the patient's laboratory results, sample menus for the meal plan, and dietary guideline for obesity-related diseases.

Participants could monitor their corresponding diseases through the app, and each disease was linked with educational videos that ran on their app. Additional educational materials could be viewed and downloaded on mobile phones running the Android operating system (Google) linked with the study app called My Health Diary. Within the app, each disease was also linked with corresponding nutritional information and recommendations. We provided general nutritional information about macronutrients, a low-salt diet, and low-calorie tips for shopping or dining out. We also provided dietary guidelines for specific diseases, such as dyslipidemia, diabetes, fatty liver, and hypertension, to patients in need (Figure 2). We presented sample menus of healthy meals for 1week in accordance with the calorie targets prescribed by a dietitian (Figure 2).

#### Personal Goal Setting, Self-monitoring, and Automatic Feedback on Weight and Physical Activities Using an Accelerometer

Participants could set their final weight goal and monthly weight goal. If they did not enter their goal, a default set of goals was automatically chosen: 10% reduction of their current weight as the final goal and a 2-kg reduction as the monthly goal. If the monthly goal was >5% of their current weight, information regarding the risk of rapid weight loss and advice for adjusting the target goal was provided.

If participants set their goal, the difference between their current weight and their target weight was automatically calculated in calories. Assuming participants adhered to their prescribed calorie targets, the app suggested daily and weekly target activity calories. Participants could select the target activity calories within a 10% range.

A wristband-type, 3-axis accelerometer (LG LifeGram, LG Electronics, Seoul, Republic of Korea) monitored the patients’ physical activity. This accelerometer analyzes the number of steps, distance, activity time, and intensity of the activity and calculates the calories burned by each activity on the basis of patients’ personal information, such as height, weight, age, and sex. We chose this accelerometer because it allowed us to use the database directly linked with the study app. Unfortunately, no studies regarding the validity of the LG LifeGram have been published.

**Figure 3:**
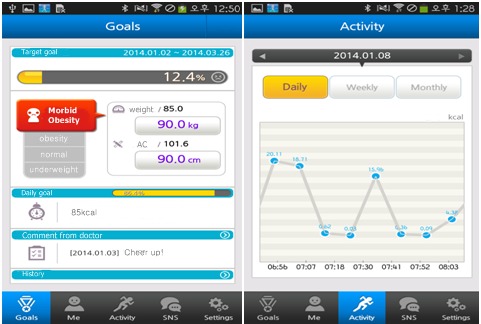
My Health Diary screenshot: regular monitoring reports include weight changes, weight goals, physical activities undertaken, and encouraging messages.

Physical activities were monitored daily by the accelerometer, which synchronized with the mobile app. Automatic feedback messages were sent to patients in accordance with the protocols based on their monthly target weight loss goal, which included the recommended calorie consumption per day, target calories burned by activities, and previous physical activity records. If the accelerometer and the mobile app were not synchronized over a 24-hour period, an automated reminder message was sent to patients. Self-reporting of daily weight was encouraged. If no weight was reported for more than 2 days, an automated reminder message was sent to patients. As Figure 3 shows, weekly graphical reports provided weight and physical activity summaries with messages of encouragement and assigned the goals for the next week.

#### Development of a Web-Based Communication Channel Between Patient and Doctor

**Figure 4:**
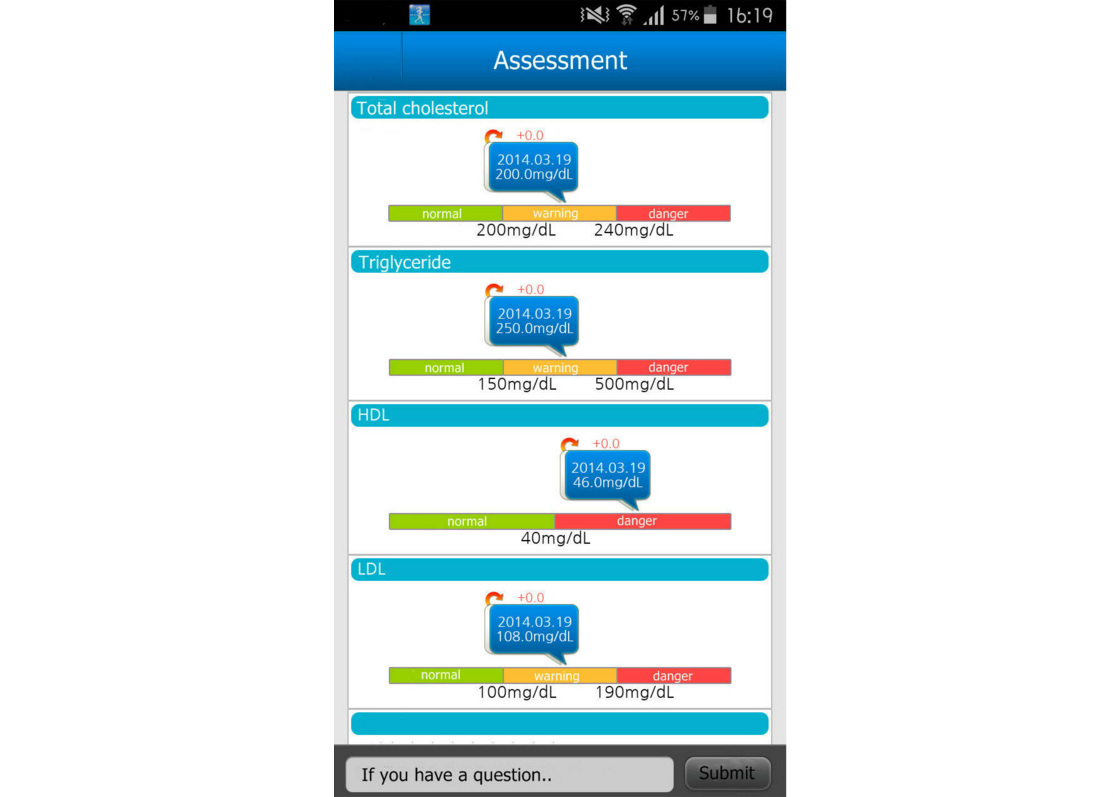
My Health Diary screenshot: message board between doctors and patients.

We developed an interactive messaging board between doctors and patients. If a patient asked questions regarding their health status or condition via the message board, doctors received an automated message as Figure 4 shows. The message board was developed to promote effective and efficient communication, which would supplement the short interview time available during clinic visits. Doctors had a choice of answering questions either via the mobile app or directly during a scheduled clinic visit with patients. Doctors could also leave encouraging messages or target goals on the message board, and messages were sent to the corresponding patient’s mobile app. The message board was developed for one-to-one chatting with each patient’s doctor, and patients could not see the contents of other participants’ messages. On the doctor’s app screen, the message board looked like a list of emails from participants, but only one-to-one communication was possible so that the participants’ sensitive medical information remained secure.

#### Peer Support Group Using a Social Network Service

**Figure 5:**
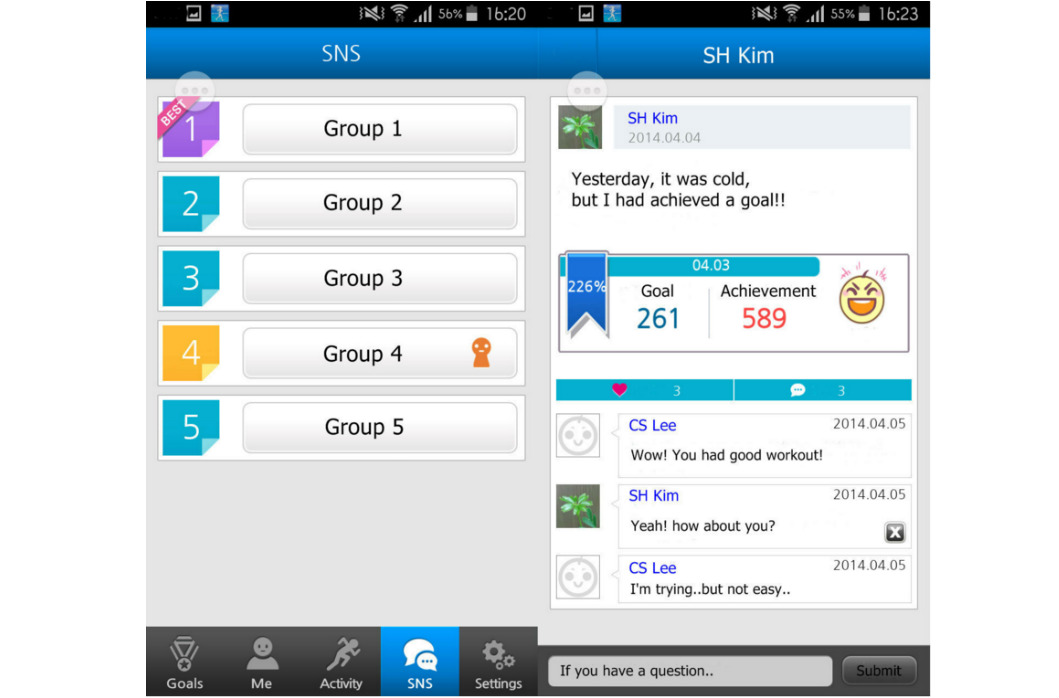
My Health Diary screenshot: social networking service for peer support group. (Fictional names entered for demonstration purposes.).

We set up a peer support group using a social networking service that was incorporated into our mobile app (Figure 5) that the participants could join. Participants could view each other’s physical activities and the percentage of their goals that they had completed, and they could communicate with each other via a peer-to-peer messaging system. Participants’ physical activities with their achievement goals were linked with the social networking service and viewable by other people by default. The default option was that only the username generated by each participant was visible in the social network service, but it was possible for the participants to upload their photo.

### Study Design

During the period of March 2014 to May 2014, we applied a pre-post single-group design to evaluate the usability and acceptability, as well as the early effects on patient-doctor relationship, of our newly developed mobile app, which was linked with an accelerometer. Usability assessments measured the technical effectiveness of the mobile app by asking whether the users could easily follow the steps without making any errors. We assessed acceptability by the users’ overall experience in using the mobile app, their perceived confidence in the information, and their satisfaction with the experience. Our aim was to implement a mobile app that runs on mobile phones as a clinical program. We did not provide telephone numbers, emails, or Web-based portal services to patients. A Web-based portal was only used for researchers to summarize participant’s anthropometric measurements, laboratory measurements, and corresponding obesity-related problems.

### Flow of the Clinic-Based Weight Loss Program

We briefly outline the clinic-based weight loss program as follows.

If a patient came to the clinic, either on their own or referred from other departments, a family physician obtained the patient’s medical history, health behaviors, previous attempts at losing weight, preferences, and goals. Then, the patient was scheduled for a meeting with a dietitian and was evaluated regarding obesity-related problems, including screening questionnaires for depression and eating disorders, body composition measurements, and laboratory examinations, such as tests for glucose, cholesterol, liver function, and kidney function. The patient could be referred to specialists in accordance with the problems identified in their initial assessment. The patient then received a treatment program consisting of lifestyle-focused interventions, including a nutritional plan, targeted behavioral changes, and an exercise program. The patient was also provided with pharmacotherapy or was referred to a bariatric surgical team if their body mass index (BMI) was >35 kg/m^2^ with comorbidities, they had previously attempted lifestyle modification and pharmacotherapy without success, and they were interested in surgical treatment. A follow-up meeting with the physician was typically scheduled at 2- to 4-week intervals for the first 3 months.

### Study Population

We recruited 30 participants from patients who visited our clinic for weight loss programs. The World Health Organization’s Regional Office for the Western Pacific Region defines obesity in Asians as those with a BMI ≥25 kg/m^2^ [39]. The Korean government officially uses this definition when defining and implementing health policies regarding obesity in Korea.

If patients met the following criteria, they were eligible for participation: BMI of ≥25 kg/m^2^, possession of an Android mobile phone, and between 20 and 70 years of age. Patients were excluded if they had been admitted to a hospital due to a cardiovascular disease in the previous 6 months, being immobile due to surgical procedures in the previous 3 months, being pregnant, having experienced weight fluctuations of >10% in the previous year, or having a history of alcohol use disorder, an eating disorder, or of using weight loss pills in the previous 3 months.

Patients who were eligible were identified and informed about the study. On their second visit to the clinic, patients were invited to participate and discuss their results from the initial assessment. If a patient agreed to participate and provided written informed consent, he or she was scheduled to contact a research staff member for instructions regarding the process of downloading, authenticating, and using the app, as well as other pertinent information, including receiving manuals and explanations for the accelerometer. Moreover, patients were followed up 4 weeks after downloading the study app.

We also explained the delay of pharmacotherapy or surgical treatment for the 1 month of the study period so that we could perform pilot testing of the app’s effect on weight changes. Patients received the equivalent of US $20 per visit as reimbursement. The study was approved by the Institutional Review Board of Seoul National University Bundang Hospital (B-1402-238-005).

### Outcome Measures

The primary outcomes measured were the acceptability of our mobile app linked with an accelerometer and changes in the patient-doctor relationship after the intervention. We measured the usability of our mobile app linked with an accelerometer via a 5-point Likert scale by asking whether the users could easily follow every step without making errors and successfully complete all steps. We analyzed usage information of each module in our mobile app as follows. (1) mean number of log-ins per day: the mean number of log-ins to the mobile app per day during the study period, which represented how often a participant used the app in a day, (2) use of each module: the number of clicks per specific module divided by the total number of clicks in the mobile app during the study period, which represented how often a participant used a specific module in this app, (3) mean exercise calories per week: the mean exercise calories per week was recorded by an accelerometer during the study period, which represented the actual exercise activities recorded by an accelerometer in a week, (4) mean percentage of weekly exercise goals achieved: the mean percentage of actual exercise calories divided by suggested exercise goals per week, which represented the amount of exercise a participant achieved in a week, (5) total numbers of messages posted on the social network services: the numbers of messages, including replies participants posted on the social network services, which represented how frequently a participant used the social network services.

We evaluated the patient-doctor relationship by the scores of the Patient-Doctor Depth-of-Relationship Scale [40]. This scale is an 8-item self-completed questionnaire for measuring 4 elements: knowledge, trust, loyalty, and respect. The survey instrument has good reported reliability (Cronbach alpha=.93).

The secondary outcomes were changes in weight and abdominal circumferences from before to after the study. A research nurse measured these when patients visited the clinic.

### Protecting Patient Privacy and Security When Using the Mobile App

According to the Personal Information Protection Act in Korea, unique identifiers, social security numbers, and bioinformation should be safely encrypted when they are intended to be transferred via a network or subsidiary storage devices. In our mobile app, we stored all individual information on the internal server, where a firewall was installed to block unauthorized access. We also incorporated an additional layer of authentication using an email address and a password.

### Statistical Analysis

We used descriptive statistics to summarize the baseline characteristics of the study participants. We analyzed the information on use for each functional category in our mobile app along with the proportion of patients who achieved their first month’s target goal. For the acceptability tests, we present the percentages for each response. Changes in weight and abdominal circumferences were calculated using a paired *t* test, and changes in the patient-doctor relationship scales were tested using a Wilcoxon signed rank test. We performed all statistical analyses using Stata version 12.1 (StataCorp LP) and considered *P<*.05 to be statistically significant.

## Results

### Baseline Characteristics of Study Participants

A total of 30 individuals agreed to participate in the study. At 1 month, 93% (28/30) of participants completed their follow-up surveys. Although 2 participants used the mobile app for 1 month, they were unable to attend their 1-month follow-up visit. Among the 30 participants, 60% (18/30) were male. Participants had a mean age of 49.07 (SD 8.84) years and a mean BMI of 27.64 (SD 2.14) kg/m^2^; 60% (18/30) of participants had dyslipidemia, 27% (8/30) had hypertension, and 10% (3/30) had diabetes mellitus. (Table 1)

### Mobile App Use Information During the Intervention Period

All study participants used the mobile app at least once during the study period even if they did not complete the follow-up visits. Table 2 presents usage information.

Participants used the study app for a median of 1.21 log-ins per day with a maximum of 6.38 log-ins per day. The most frequently used module was goal setting (median 199 times, 74%), followed by tracking of physical activities (median 30 times, 13.5%) and viewing of personal health status (median 34.5 times, 10.8%).

Most study participants achieved more than their recommended exercise; the median percentage of target exercise goals achieved per week was 125.9%. The mean number of messages posted on the social network service was 1 during the entire study period, and only 6 patients used the social network service.

### Usability of Mobile App After 1 Month of Intervention

With regard to the app’s ease of use, participants reported a high rate of positive responses for the log-in process (25/28, 89%) and goal setting process (23/28, 82%), as presented in Table 3. However, the rate of positive responses for the message board was low, including posting (11/28, 39%) and replying (10/28, 36%) on the social network service. Additionally, the positive response rate for the educational videos was 46% (13/28).

### Acceptability of the Mobile App and Patient-Doctor Relationship Scales

As Table 4 shows, regarding participants’ satisfaction with each module of the mobile app, setting personal goals had the highest positive response rate, at 75% (21/28). Ratings of satisfaction with the monitoring and feedback on physical activities were also highly positive. However, satisfaction with the social network service, educational videos, problem solving in case of errors, and reliability of the contents was approximately 50% (14/28, 14/28, 15/28, and 15/28), which was low relative to other modules. Nonetheless, most users (26/28, 93%) responded positively to receiving help with managing weight from the mobile app. Specifically, 75% (21/28) of users were willing to recommend this app to their family or friends, and 79% (22/28) (expressed interest in continuing to use this app for weight management.

Scales measuring the depth of the patient-doctor relationship decreased during the 1-month period from 27.6 (SD 4.8) to 25.1 (SD 4.5) by a Wilcoxon signed rank test (*P*=.02).

After the 1-month study period, the mean weight change was –0.1 kg (95% CI –0.6 to 0.8) and we observed no significant changes. However, abdominal circumference was significantly reduced by a mean of –1.84 cm (95% CI –3.3 to –0.4) (see Multimedia Appendix 2).

**Table 1:** Baseline characteristics of study participants (n=30).

Variable	Result
Male, n (%)	18 (60.0)
Age in years, mean (SD)	49.07 (8.84)
Weight in kg, mean (SD)	76.82 (8.38)
Abdominal circumference in cm, mean (SD)	93.55 (4.92)
BMI^a^ in kg/m^2^, mean (SD)	27.64 (2.14)
Systolic blood pressure in mmHg, mean (SD)	111.07 (39.85)
Diastolic blood pressure in mmHg, mean (SD)	70.70 (25.40)
Fasting glucose in mg/dL, mean (SD)	86.87 (37.24)
Triglyceride in mg/dL, mean (SD)	121.90 (80.08)
HDL^b^ cholesterol in mg/dL, mean (SD)	42.8 (20.17)
LDL^c^ cholesterol in mg/dL, mean (SD)	102.97 (56.05)
Uric acid in mg/dL, mean (SD)	5.20 (2.59)
Hypertension, n (%)	8 (26.7)
Diabetes mellitus, n (%)	3 (10.0)
Dyslipidemia, n (%)	18 (60.0)
Ischemic heart disease, n (%)	1 (3.3)
Cerebrovascular disease (n, %)	1 (3.3)
Gastroesophageal reflux disease, n (%)	7 (23.3)
Obstructive sleep apnea, n (%)	5 (16.7)

^a^BMI: body mass index.

^b^HDL: high-density lipoprotein.

^c^LDL: low-density lipoprotein.

**Table 2:** Study participants’ use of each module of the mobile app for weight management in the clinic (n=30).

Aspect of use		Minimum	25^th^ percentile	Median	Mean	75^th^ |percentile	Maximum
Mean number of log-ins per day^a^		0.03	0.73	1.21	1.88	2.65	6.38
**Numbers of clicks on each module, n (%)** ^b^							
	Goal setting	24.0 (36.0)	151.0 (61.5)	199.0 (74.4)	217.13 (71.3)	290.0 (77.9)	549.0 (98.8)
	My personal health status	1 (0.6)	21.0 (8.9)	34.50 (10.8)	40.83 (12.3)	52.0 (16.0)	136.0 (33.7)
	Educational videos and nutritional information	0.0 (0)	2 (0.9)	5.5 (1.9)	6.6 (2.1)	10.0 (2.8)	19.0 (6.7)
	Tracking my physical activities	1 (0.6)	20.0 (9.6)	30.0 (13.5)	55.7 (14.3)	69.0 (18.4)	285.0 (31.9)
Mean exercise calories per week^c^		46.0	1740.0	2575.0	2612.8	3211.5	7125.5
Mean percentage of weekly exercise goals achieved (%)^d^		3.4	87.9	125.9	141.1	167.1	601.7
Total numbers of messages posted on patient-doctor communication board		0	0	0	0.5	1	3
Total numbers of messages posted on social network service^e^		0	0	0	1	0	10

^a^The number of days that patients logged into the mobile app at least once during the study period.

^b^Usage rate of each module is calculated as the percentage of the number of clicks on each module divided by the total number of clicks on all mobile app menus during the study period.

^c^The mean calories expended in exercise per week recorded by the accelerometer during the study period.

^d^The mean percentage of actual exercise calories divided by suggested exercise goals per week.

^e^The numbers of messages including replies that participants posted on the social network service.

**Table 3:** Number of responses regarding usability of a mobile app for weight management in the clinic (n=28).

Aspect of usability	Strongly agree	Agree	Neutral	Disagree	Strongly disagree
Log-in process	7	18	1	1	1
Goal setting	6	17	3	1	1
My personal health status	3	15	9	1	0
Educational videos and nutritional information	1	12	11	3	1
Tracking my physical activities	4	13	10	1	0
Posting comments on social network	1	10	15	2	0
Replying to comments on social network	2	8	16	2	0
Sharing information regarding physical activities through social network	3	11	13	1	0
Ease of learning each function	2	17	8	1	0

**Table 4:** Number of responses regarding acceptability of a mobile app for weight management in the clinic (n=28).

Aspect of acceptability		Strongly agree	Agree	Neutral	Disagree	Strongly disagree
**Satisfaction**						
	Goal setting	4	17	6	1	0
	Educational videos	1	13	12	2	0
	Nutritional information	3	13	12	0	0
	Social network service	3	11	13	1	0
	Tracking physical activities	7	13	7	0	1
	Feedback on physical activities	6	14	5	2	1
Contents in mobile app		8	13	7	0	0
Convenience in using mobile app		5	15	8	0	0
Integration of each function		2	13	11	2	0
Font size and styles		4	15	8	1	0
Response to problems with using the app		2	13	12	1	0
Reliability of contents		3	12	13	0	0
Helpfulness in managing weight		9	17	2	0	0
Likelihood of recommending this app to family or friends		7	14	7	0	0
Continuous use		10	12	5	1	0

## Discussion

To our knowledge, this was the first study to test the usability and acceptability of a mobile app linked with an accelerometer for a clinic-based weight loss program, and was also the first to evaluate such an app’s early effect on the patient-doctor relationship. Most participants used our mobile app during the 1-month study period, and the goal setting function with self-monitoring of physical activities linked with an accelerometer was the most frequently used module. As this was a pilot study for the purpose of further improving our mobile app, we focused on the usability and acceptability of our app, as well as on the preliminary findings concerning the patient-doctor relationship.

### Development of a Mobile App Linked With an Accelerometer for a Clinic-Based Weight Management Program

In contrast with many other weight loss apps that target the general population as a health and fitness tool, our mobile app was targeted to the population that participates in a clinic-based weight management program. Our choice of target population was based on several factors that are particular to hospital settings.

Numerous different phenotypic presentations [41,42] and risks that affect obesity-related mortality have been noted [43], even within the same BMI. Therefore, recognizing obesity-related comorbidities and functional status is essential for treatment planning, as well as for prescribing exercise or nutrition. These evaluations are typically possible only in a clinic or hospital setting with a multidisciplinary health care team. Hence, management of weight loss should not only target weight loss itself but also aim at controlling obesity-related health problems. Consequently, a commercially available weight loss app cannot deliver tailored information to each patient. We attempted to incorporate medical information from EHRs to deliver the appropriate information to each patient in need via tailored education videos and nutritional information. Moreover, bariatric surgery continues to be the most effective method for initiating significant improvement in obesity-related comorbidities [44,45], and the need for multidisciplinary weight management is increasing in hospital settings for both pre- and postoperational care. Therefore, mobile technology can contribute to a precise assessment of obesity-related comorbidities, thereby enhancing the medical and surgical treatments.

Given that informed, active patients, or patients who have the motivation, knowledge, skill, and confidence, play a large role in improving their own health outcomes [46], as well as reducing health care costs [47], our mobile app could have potential use as a tool for supporting patient activation in hospitals by providing tailored education along with self-management skills.

A mobile app linked with an accelerometer could be a good resource for clinicians for monitoring patient behaviors or physiological signals. In addition, if such information is recognized and used during a clinic or hospital visit, high-quality doctor-patient exchanges both inside and outside of the office visit can be achieved, which could favorably influence patients’ behavior and attitude [24].

Although our study did not use EHR-linked personal health records, our methods can be applied to incorporating them in chronic disease management, where patients require constant care and regular follow-ups.

### Usability and Acceptability of the Mobile App

Based on app usage data, the most frequently used module was goal setting, as well as the monitoring of and feedback on physical activities, which was concordant with the subjective satisfaction measures reported in the questionnaires. The median number of log-ins per day was 1.2, and all participants used our app over the course of the 1-month intervention period. Furthermore, participants achieved more than their suggested activity amount. We linked an accelerometer to our mobile app automatically, which potentially contributed to its frequent use and the successful achievements within our app. The self-regulation function was the main feature of our program, and participants evaluated this feature as the most comfortable and helpful through both objective and subjective measurements. These results were consistent with other studies [48,49], which further validates the benefit of mobile health apps in increasing physical activity. This increase may also result in a reduction in the abdominal circumferences of study participants by the end of the study period.

Conversely, we only provided nutritional guidelines that matched with the metabolic profiles of each participant, and we did not track nutrition, which may have affected the weight loss outcomes. Although abdominal circumferences were reduced by a mean of -1.84 cm during the study period, we noted no weight change. Research has demonstrated that frequent recording of dietary intake can contribute to weight loss [50]. However, recording food intake has been the most laborious and challenging work in mobile phone apps or websites for weight loss [51]. Healthy diet education via a mobile app did not affect weight loss. However, eliminating the food diary, which is time consuming, might also lead to a low attrition rate for mobile apps. Furthermore, a food recording function that is user friendly and less time consuming is necessary for achieving desirable outcome in a clinic-based weight loss program.

The satisfaction rate was low for the personalized mobile education module. We attempted to provide the study participants with tailored educational videos. Most participants did not download the educational videos from an Android operating system even if they received instructions on how to download the videos. The size of each educational video was between 31.8 and 52.4 MB. Therefore, the process of downloading and viewing educational videos may have been too difficult or too time consuming, or patients may have simply been in an area with no Internet or network connection.

Moreover, only a few participants used the social network service. Although social networking has been used extensively in intervention approaches, including those targeted at weight loss, no clear effect of social networking on weight change has been reported in randomized controlled trials [52]. However, participants received more encouragement from the community in many weight loss intervention programs that used social network services [52]. The low usage of this module combined with low satisfaction among our study participants may be attributed to several factors. The patients were all new users to our social network service, and no one had adequate experience to act as a facilitator. They study intervention period was only 1 month long, hindering the formation of a strong support group. Given the age and sex of our study participants, patients might not have been exposed to online social networking in general. The contents of the online networking in this study app might not have met participants’ needs, or the usability might not have been optimal, which is important for both user engagement and behavioral change [53]. However, research into the theory and function of online social networking and its impact on health outcomes is still in early development, and more studies regarding the actual usability combined with the contextual factors of social networking are needed. According to a systematic review [54], most studies reported that online social networking in attempts to change health behavior had poor retention and engagement.

### Patient-Doctor Relationship Scales

Our mobile app had a unique feature: an interactive message board that was used to help patients share information with their doctors. However, according to the usage analysis, only a few participants used the interactive message board for communication. Family physicians in charge of patients left at least 2 messages for all participants under their care: 1 at the beginning of the study period to encourage behavioral change, and the other 2 weeks later to address questions about the weight loss program or process. Only 2 patients sent messages to their doctors: 1 pertained to how to take care of high blood pressure measured at home, and the other pertained to medication for controlling diabetes. Doctors replied to the participant’s question via the message board, but the participants were informed that the doctor’s reply may be instant or provided at the time of their scheduled session. Such misinformation may have inhibited the participants from using the interactive message board.

In our study, scales measuring the depth of the patient-doctor relationship decreased during the 1-month study period. This effect could reflect several aspects related to using the mobile app in a clinical setting. First, the doctor’s passive participation in the app, particularly in the interaction with a patient, might have been a factor in the decreased depth in the patient-doctor relationship. Second, the doctor’s encouragement or feedback regarding app usage might not have been recognized during the clinic visit, or the doctor’s feedback might not have been sufficiently positive. According to one survey regarding information technology and its impact on doctor-patient communication [55], panelists, including both medical professionals and patient advocates, expected improvement in the relationship. However, the lack of acceptance by doctors and issues of data security and monetary costs were regarded as barriers. Third, patient-doctor relationship scales might not adequately reflect short-term changes. Patient-doctor relationship scales were designed to measure the quality of the relationship in the context of continuity of care, whereas our study period was only 1 month. Fourth, empowering patients with self-management skills could make the patient-doctor relationship more complicated, and this phenomenon could have a negative effect on the short-term patient-doctor relationship if a doctor did not exhibit a supportive attitude toward using the apps. Moreover, cultural aspects of the relationship between a patient and his or her doctor in Korea may have played a role in preventing any improvement [56].

### Study Limitations

This study had several limitations. It was a pilot study of a single group that used a pre-post intervention design, which only demonstrated the possibilities for further improvement and clarification of the app’s design and treatment functions. The research was performed in 1 clinic for a limited time using a small number of participants, which may not reflect the diverse population of patients with weight problems. We attempted to implement the function of self-monitoring of physical activity by synchronizing the accelerometer with the app, but LG LifeGram has not published its validity test, which might affect the usage pattern for this study app.

However, to our knowledge, this study is one of the first to demonstrate the usability and acceptability of a mobile app linked with an accelerometer for a clinic-based weight loss program and its potential impact on the doctor-patient relationship. The mean age of the study participants was 49.07 years, which is generally older than other mobile app studies [30,57] with a greater proportion being male participants. This finding could potentially indicate that these were less-active users for a mobile weight loss app. However, all participants used the mobile app during the study period, achieved their suggested physical activity goals, and reported a high rate of satisfaction with the self-regulation function. One study [58] suggested that men would be willing to use Web-based self-monitoring tools delivered by mobile phones if interventions were easy, quick, and simple to use but showed little interest in using the social networking service. Our study results confirm these observations to some degree. Further studies identifying a mediating effect of age and sex will be needed to deliver a tailored intervention through a mobile app.

This mobile app has the potential for furthering improvements in achieving weight loss if it includes more strategies for reaching dietary goals, improvements in pattern monitoring and stress management techniques [59], and improvements in its structure, ease of use, personalized features, and accessibility in technology and design [60]. Further studies with a larger representative population and longer duration are needed.

### Conclusions

In this study, we evaluated a mobile app linked with an accelerometer for a clinic-based weight loss program. Mobile health apps have the potential to be integrated into a clinic-based weight management program by empowering patients with self-regulation tools that enhance physical activity.

An easier method for recording daily food intake should be incorporated into the mobile app for better weight loss outcomes. Further reflection of the user’s needs with regard to the online social network and the patient-doctor relationship should also be considered.

## Appendix

Multimedia Appendix 1 The educational video for angina pectoris.

Multimedia Appendix 2 Changes in weight and abdominal circumference after a 1-month intervention.

## Abbreviations

BMI: body mass index
EHR: electronic health records
