# Supplementary material for: Development of a Weight Loss Mobile App Linked With an Accelerometer for Use in the Clinic: Usability, Acceptability, and Early Testing of its Impact on the Patient-Doctor Relationship
Source: JMIR Mhealth Uhealth. 2016 Mar 31;4(1):e24. doi: 10.2196/mhealth.4546 (PMC4832121; doi:10.2196/mhealth.4546)
Supplement: Multimedia Appendix 2 [file mhealth_v4i1e24_app2.pdf]

Multimedia Appendix 2

Table 1. Changes in weight and abdominal circumference after a 1 month intervention with a mobile app for weight management in the clinic (n = 28)

	Baseline	After intervention	Difference	95% CI	*P*-value
Weight, kg (mean ± SD)	76.79 ± 8.51	76.90 ± 8.61	0.12	(-0.59, 0.82)	0.73
AC[a], cm (mean ± SD)	93.59 ± 5.04	91.75 ± 4.96	-1.84	(-3.26, -0.41)	0.01

[a]AC: abdominal circumference

[b]mean±SD based on descriptive statistics

[c]tested using a Wilcoxon signed rank test.
